# Anti-Diabetic Effects of Madecassic Acid and Rotundic Acid

**DOI:** 10.3390/nu7125512

**Published:** 2015-12-02

**Authors:** Yuan-Man Hsu, Yi-chih Hung, Lihong Hu, Yi-ju Lee, Mei-chin Yin

**Affiliations:** 1 Department of Biological Science and Technology, China Medical University, Taichung City 40402, Taiwan; yuanmh@mail.cmu.edu.tw; 2 Graduate Institute of Clinical Medical Science, China Medical University, Taichung City 40402, Taiwan; viennaspring0312@gmail.com; 3 Division of Endocrinology and Metabolism, Department of Internal Medicine, China Medical University Hospital, Taichung City 40402, Taiwan; 4 Shanghai Research Center for the Modernization of Traditional Chinese Medicine, Shanghai Institute of Materia Medica, Chinese Academy of Sciences, Shanghai 201203, China; lhhu@simm.ac.cn; 5 Department of Pathology, Chung Shan Medical University Hospital, Taichung City 40402, Taiwan; jasmine.lyl@gmail.com; 6 Department of Health and Nutrition Biotechnology, Asia University, Taichung City 40402, Taiwan; 7 Department of Nutrition, China Medical University, Taichung City 40402, Taiwan

**Keywords:** madecassic acid, rotundic acid, diabetes, coagulation, anti-lipid

## Abstract

Anti-diabetic effects of madecassic acid (MEA) and rotundic acid (RA) were examined. MEA or RA at 0.05% or 0.1% was supplied to diabetic mice for six weeks. The intake of MEA, not RA, dose-dependently lowered plasma glucose level and increased plasma insulin level. MEA, not RA, intake dose-dependently reduced plasminogen activator inhibitor-1 activity and fibrinogen level; as well as restored antithrombin-III and protein C activities in plasma of diabetic mice. MEA or RA intake decreased triglyceride and cholesterol levels in plasma and liver. Histological data agreed that MEA or RA intake lowered hepatic lipid droplets, determined by ORO stain. MEA intake dose-dependently declined reactive oxygen species (ROS) and oxidized glutathione levels, increased glutathione content and maintained the activity of glutathione reductase and catalase in the heart and kidneys of diabetic mice. MEA intake dose-dependently reduced interleukin (IL)-1β, IL-6, tumor necrosis factor-α and monocyte chemoattractant protein-1 levels in the heart and kidneys of diabetic mice. RA intake at 0.1% declined cardiac and renal levels of these inflammatory factors. These data indicated that MEA improved glycemic control and hemostatic imbalance, lowered lipid accumulation, and attenuated oxidative and inflammatory stress in diabetic mice. Thus, madecassic acid could be considered as an anti-diabetic agent.

## 1. Introduction

**Figure 1:**
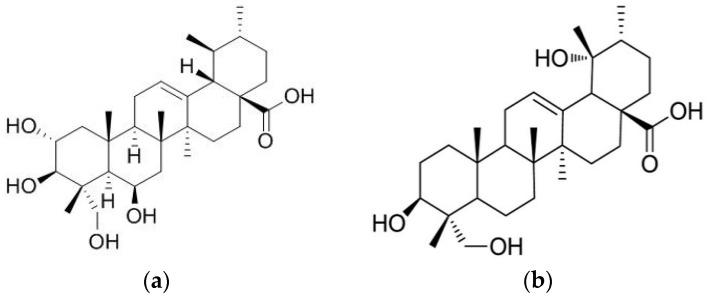
Structure of madecassic acid and rotundic acid. (**a**) Madecassic Acid; (**b**) Rotundic Acid.

Madecassic acid (MEA) and rotundic acid (RA) are pentacyclic triterpenic acids (Figure 1). MEA is a major triterpenic acid present in *Centella asiatica* [1], and RA occurs in *Mussaenda macrophylla* and *Glochidion obliquum* [2,3]. Won *et al*. (2010) reported that MEA could attenuate lipopolysaccharides induced inflammatory stress in RAW 264.7 macrophage cells via suppressing nuclear factor kappa B pathway [4]. The study of Tabassum *et al*. (2013) suggested that MEA contributed to the anti-oxidative protection of ethanolic extract prepared from *C. asiatica* in cerebral artery occlusion rats [5]. Our previous study revealed that MEA could be detected in some edible plants including gynura (*Gynura bicolor* DC.), basil (*Ocimum basilicum*), and daylily (*Hemerocallis fulva* L.), and its content was in the range of 11–73 mg/100 g dry weight [6]. Furthermore, we found that dietary intake of MEA enhanced anti-oxidative defense in mice hearts and kidneys [6]. Zhang *et al*. (2014) indicated that MEA was able to inhibit mouse colon cancer growth [7]. Thang *et al*. (2011) found that RA displayed anti-inflammatory effects in murine microglial cells [8]. The cytotoxic activities of RA toward human hematoma cell line (HepG2), malignant melanoma cell line (A375), breast cancer cell line (MCF-7), and colon cancer cell line (HT-29) have been observed [9,10]. Although those previous studies suggest that MEA and RA are natural compounds with several bio-activities, less information is available regarding the *in vivo* protective activities of MEA or RA against diabetes.

Poor glycemic control is an important factor responsible for the progression of diabetic complications, which exacerbate the severity and mortality of this disease [11]. The clinical characteristics of diabetic complications include coagulation predomination, hyperlipidemia, oxidative stress, and cytokine imbalance [12,13,14]. The increase of blood coagulation factors such as fibrinogen and plasminogen activator inhibitor (PAI)-1, and decrease of anti-coagulation factors such as antithrombin III (AT-III) and protein C occurred in diabetic patients cause hypercoagulability, and favor the development of thrombosis, stroke, myocardial infarction, and/or glomerulosclerosis [15,16]. Thus, besides glycemic control, hemostatic imbalance warrants attention in order to retard diabetic deterioration. So far, it remains unknown that MEA or RA could alter coagulatory and anti-coagulatory factors. 

Hyperlipidemia including hypertriglyceridemia and hypercholesterolemia in diabetes, mainly due to insulin deficiency, raises the prevalence of premature atherosclerosis [17]. The excessive production of reactive oxygen species (ROS), pro-inflammatory cytokines, and chemokines such as interleukin (IL)-1β, tumor necrosis factor (TNF)-α, and monocyte chemoattractant protein (MCP)-1 not only enhance systemic oxidative and inflammatory injury in diabetic individuals but also promote the progression of diabetes associated cardiac and/or renal disorders [18,19]. If MEA or RA could provide lipid-lowering, anti-oxidative and/or anti-inflammatory activities, they may prevent or delay the development of diabetic complications. 

In this present study, the hypoglycemic effects of MEA and RA in diabetic mice were examined. The anti-coagulatory, lipid-lowering, anti-oxidative, and anti-inflammatory effects of these two compounds were also evaluated. These results provided novel findings regarding the possible application of these triterpenic acids as anti-diabetic agents.

## 2. Materials and Methods

### 2.1. Materials

MEA (98%) and RA (98.5%) were obtained from Shanghai Institute of Materia Medica, Chinese Academy of Sciences, China. Other chemicals were purchased from Sigma Chemical Co. (St. Louis, MO, USA). All chemicals used in these measurements were of the highest purity commercially available.

### 2.2. Animals and Diets

Male Balb/cA mice, three to four weeks old, were purchased from the National Laboratory Animal Center (Taipei City, Taiwan). Mice were housed on a 12-h light:dark schedule, water and mouse standard diet were consumed *ad libitum*. The use of mice was reviewed and approved by China Medical University animal care committee (104–305). To induce diabetes, mice with body weight of 24.1 ± 1.3 g were treated with a single i.v. dose (50 mg/kg) of streptozotocin dissolved in citrate buffer (pH 4.5) into the tail vein of 12-h fasted mice. Blood glucose level was monitored on day 10 from the tail vein using a one-touch blood glucose meter (Lifescan, Inc. Milpitas, CA, USA). Mice with fasting blood glucose levels ≥ 14.0 mmol/L were used for this study. After diabetes was induced, mice were divided into several groups (10 mice per group). 

### 2.3. Experimental Design

MEA or RA at 0.05 or 0.1 g was mixed with 99.95 or 99.9 g powder diet to prepare 0.05% and 0.1% diets, and supplied to diabetic mice. All mice had free access to food and water at all times. Body weight, consumed water volume, and feed were recorded. After six weeks of supplementation, mice were killed with carbon dioxide. Blood was collected, and plasma was separated from erythrocytes immediately. Heart, liver, and kidney were collected and weighed. Each organ at 0.1 g was homogenized on ice in 2 mL phosphate buffer saline (PBS, pH 7.2), and the filtrate was collected. The protein concentration of plasma or organ filtrate was determined by a commercial assay kit (Pierce Biotechnology Inc., Rockford, IL, USA) with bovine serum albumin as a standard. 

### 2.4. Blood Glucose and Insulin Analyses

The plasma glucose level (mmol/L) was measured by a glucose kit (Sigma Chemical Co., St. Louis, MO, USA). Plasma insulin level (nmol/L) was measured by using a rat insulin radioimmunoassay kit (Linco Research Inc., St. Charles, MO, USA). 

### 2.5. Measurement of Coagulation and Anti-Coagulation Factors

Coagulation factors, PAI-1 and fibrinogen, anti-coagulation factors, AT-III, and protein C, were measured in this study. Blood samples were anticoagulated using sodium citrate according to the protocols provided by the manufacturers. PAI-1 activity (kU/L) was assayed by a commercial kit (Trinity Biotech plc., Bray, C. Wicklow, Ireland). Fibrinogen level (g/L) was detected based on the principle of salting out by using a commercial kit (Iatroset Fbg, Iatron Laboratory, Tokyo, Japan). The activity (%) of AT-III and protein C in plasma was determined by chromogenic assay according to the manufacturer’s instruction using commercial AT-III or protein C kit (Sigma Chemical Co., St. Louis, MO, USA), and was shown as the ratio of those in normal human plasma.

### 2.6. Determination of Triglyceride (TG) and Cholesterol

TG and total cholesterol (TC) levels in plasma (g/L) were determined by commercial triglyceride kit and cholesterol kit (Boehringer Mannheim, Ingelheim am Rhein, Germany), respectively. Total lipids were extracted from liver after extraction with the mixture of methanol and chloroform [20]. Hepatic TG or TC concentration (mg/g wet tissue) was quantified by a reagent kit purchased from Wako Chem. Co. (Wako Chem. Co., Tokyo, Japan). 

### 2.7. Assay of Oxidative and Anti-Oxidative Status

ROS level was quantified by using 2′,7′-dichlorofluorescein diacetate. Fluorescence value was measured by using a fluorescence microplate reader at excitation and emission wavelengths of 485 and 530 nm, respectively. Relative fluorescence unit (RFU) was the difference in fluorescence values obtained at 0 and 5 min. Results are expressed as RFU/mg protein. Glutathione (GSH) and oxidized glutathione (GSSG) concentrations (nmol/mg protein) in the heart or kidney were determined by commercial colorimetric GSH and GSSG assay kits (OxisResearch, Portland, OR, USA). Glutathione peroxidase (GPX), glutathione reductase (GR), and catalase activities (U/mg protein) in heart or kidney were assayed by commercial kits (Calbiochem Inc., San Diego, CA, USA). 

### 2.8. Quantification of Inflammatory Cytokines

Cardiac or renal tissue was homogenized in 10 mM Tris-HCl buffered solution (pH 7.4) containing 2 M NaCl, 1 mM ethylenediaminetetraacetic acid, 0.01% Tween 80, 1 mM phenylmethylsulfonyl fluoride, and centrifuged at 9000× *g* for 30 min at 4 °C. The resultant supernatant was used for cytokine determination. The levels of IL-1β, IL-6, TNF-α, and MCP-1 were measured by ELISA using cytoscreen immunoassay kits (BioSource International, Camarillo, CA, USA). Samples were assayed in duplicates according to manufacturer’s instructions. 

### 2.9. Histological Analyses

Partial liver tissue from each mouse was fixed in 10% phosphate-buffered formalin, and embedded in paraffin. A paraffin section was cut at 5 μm thickness and stained with Oil Red O (ORO) stain, and examinined under a light microscope for histological analyses. 

### 2.10. Statistical Analyses

All data were expressed as mean ± standard deviation (SD). Statistical analysis was done using one-way analysis of variance and post-hoc comparison was carried out using Dunnett’s *t*-test. Statistical significance is defined as *p* < 0.05.

## 3. Results

### 3.1. MEA Improved Glycemic Control and Hemostatic Imbalance 

**Figure 2:**
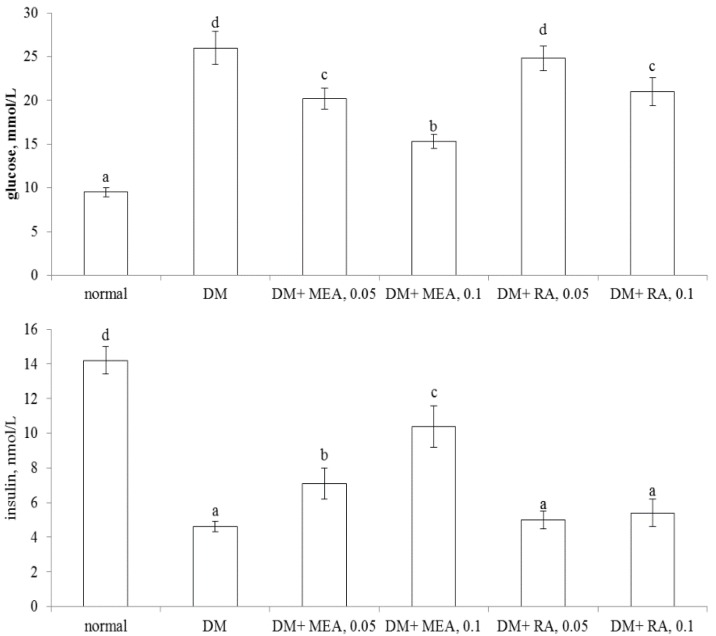
Plasma levels of glucose (mmol/L, upper part) and insulin (nmol/L, lower part) of normal (non-diabetic), diabetic mice consumed normal diet (DM), or 0.05%, 0.1% MEA or RA for six weeks. Data are mean ± SD, *n* = 10. a–d Means among bars without a common letter differ, *p* < 0.05.

As shown in Table 1, MEA intake at both doses decreased water intake and feed intake, raised body weight in diabetic mice when compared with diabetic control groups (*p* < 0.05). RA intake only at high dose (0.1%) decreased feed intake and increased body weight (*p* < 0.05). MEA and RA intake did not change heart weight, liver weight, and kidney weight in diabetic mice (*p >* 0.05). MEA intake dose-dependently lowered plasma glucose level and increased plasma insulin level (Figure 2, *p* < 0.05). RA intake only at high dose reduced plasma glucose level (*p* < 0.05) and failed to alter insulin level (*p* > 0.05). As shown in Table 2, MEA intake dose-dependently reduced PAI-1 activity and fibrinogen level, as well as restored AT-III and protein C activities in diabetic mice (*p <* 0.05). RA intake did not affect these coagulatory and anti-coagulatory factors in diabetic mice (*p* > 0.05).

**Table 1:** Water intake (WI, mL/mouse/day), feed intake (FI, g/mouse/day), body weight (BW, g/mouse), heart weight (HW, mg/mouse), liver weight (LW, mg/mouse) and kidney weight (KW, mg/mouse) of normal (non-diabetic), diabetic mice consumed normal diet (DM), or 0.05%, 0.1% MEA or RA for six weeks. Data are mean ± SD, *n* = 10.

	Normal	DM	DM + MEA, 0.05	DM + MEA, 0.1	DM + RA, 0.05	DM + RA, 0.1
WI	2.2 ± 0.6 ^a^	6.8 ± 1.4 ^d^	5.7 ± 1.0 ^c^	3.9 ± 0.8 ^b^	6.4 ± 0.7 ^d^	6.2 ± 0.5 ^d^
FI	2.4 ± 0.9 ^a^	7.0 ± 1.3 ^d^	5.2 ± 1.1 ^c^	3.6 ± 0.5 ^b^	6.5 ± 0.9 ^d^	5.5 ± 0.6 ^c^
BW	29.5 ± 1.3^d^	12.2 ± 1.0 ^a^	15.1 ± 0.8 ^b^	19.4 ± 1.2 ^c^	13.1 ± 1.3 ^a^	14.7 ± 0.7 ^b^
HW	275 ± 11 ^a^	269 ± 9 ^a^	270 ± 6 ^a^	281 ± 10 ^a^	272 ± 12 ^a^	277 ± 8 ^a^
LW	1466 ± 18 ^a^	1452 ± 23 ^a^	1455 ± 19 ^a^	1460 ± 15 ^a^	1456 ± 18 ^a^	1462 ± 13 ^a^
KW	453 ± 11 ^a^	448 ± 14 ^a^	445 ±10 ^a^	457 ± 16 ^a^	442 ± 8 ^a^	450 ± 12 ^a^

^a–d^ Means in a row without a common letter differ, *p* < 0.05.

**Table 2:** Coagulatory factors, PAI-1 activity (kU/L) and fibrinogen level (g/L); anti-coagulatory factors, AT-III activity (%) and protein C activity (%) in plasma from normal (non-diabetic), diabetic mice consumed normal diet (DM), or 0.05%, 0.1% MEA or RA for 6 wks. Data are mean ± SD, *n* = 10.

	Normal	DM	DM + MEA, 0.05	DM + MEA, 0.1	DM + RA, 0.05	DM + RA, 0.1
PAI-1	7.5 ± 0.8 ^a^	17.9 ± 1.1 ^d^	13.9 ± 1.0 ^c^	10.4 ± 0.9 ^b^	17.5 ± 1.5 ^d^	16.9 ± 1.3 ^d^
Fibrinogen	2.18 ± 0.08 ^a^	4.81 ± 0.22 ^d^	4.03 ± 0.12 ^c^	3.24 ± 0.13 ^b^	4.72 ± 0.09 ^d^	4.68 ± 0.17 ^d^
AT-III	129 ± 9 ^c^	70 ± 7 ^a^	92 ± 5 ^b^	117 ± 4 ^c^	72 ± 3 ^a^	75 ± 6 ^a^
Protein C	101 ± 8 ^c^	66 ± 5 ^a^	81 ± 3 ^b^	96 ± 6 ^c^	68± 4 ^a^	70 ± 7 ^a^

^a–d^ Means in a row without a common letter differ, *p* < 0.05.

**Table 3:** Level of triglyceride (TG) and total cholesterol (TC) in plasma (g/L) and liver (mg/g wet tissue) from normal (non-diabetic), diabetic mice consumed normal diet (DM), or 0.05%, 0.1% MEA or RA for six weeks. Data are mean ± SD, *n* = 10.

	Normal	DM	DM + MEA, 0.05	DM + MEA, 0.1	DM + RA, 0.05	DM + RA, 0.1
Plasma						
TG	2.06 ± 0.14 ^a^	3.98 ± 0.27 ^d^	3.21 ± 0.13 ^c^	2.67 ± 0.15 ^b^	3.26 ± 0.1 ^c^	2.58 ± 0.17 ^b^
TC	1.31 ± 0.1 ^a^	2.69 ± 0.13 ^d^	2.13 ± 0.12 ^c^	1.76 ± 0.11 ^b^	2.21 ± 0.08 ^c^	1.82 ± 0.16 ^b^
Liver						
TG	25.1 ± 2.3 ^a^	52.3 ±4.2 ^e^	41.4 ± 2.2 ^c^	34.7 ± 1.4 ^b^	46.5 ± 2.0 ^d^	40.3 ± 3.1 ^c^
TC	3.46 ±0.18^a^	5.62 ± 0.31 ^d^	4.93 ± 0.22 ^c^	4.18 ± 0.16 ^b^	5.05 ± 0.18 ^c^	4.87 ± 0.19 ^c^

**Figure 3:**
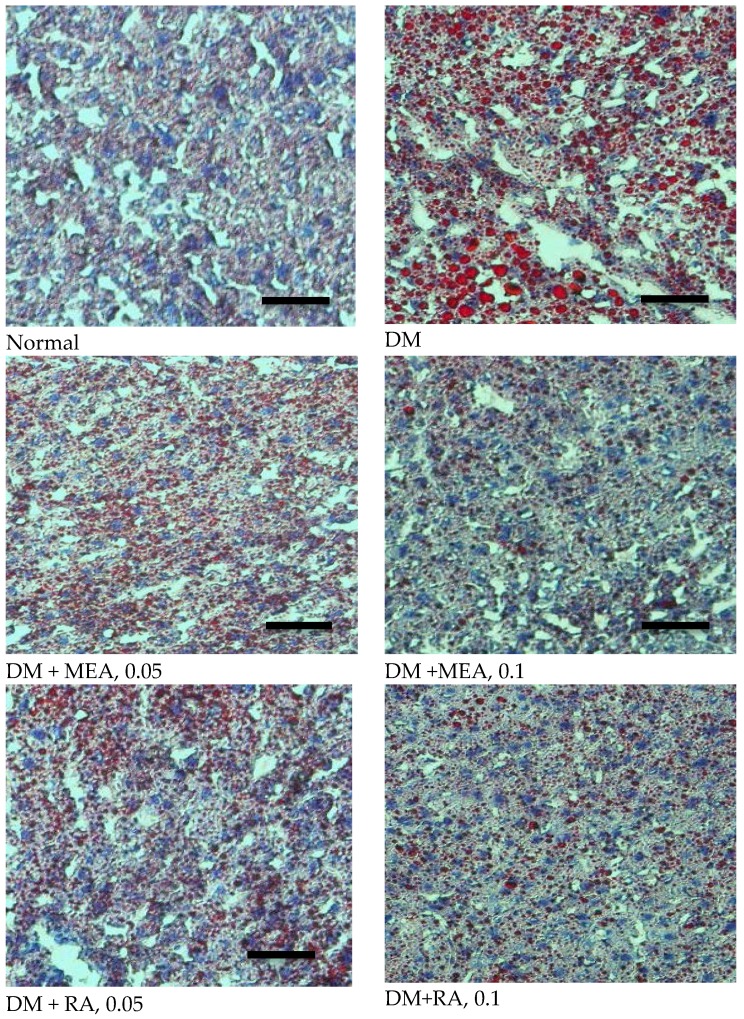
Effects of MEA and RA upon hepatic lipid accumulation, determined by ORO statin in normal (non-diabetic), diabetic mice consumed normal diet (DM), or 0.05%, 0.1% MEA or RA for six weeks. A representative image is shown for each group. Scale bar: 50 μm. Magnification: 200×.

### 3.2. MEA and RA Lowered TG and TC Levles

As shown in Table 3, diabetes led to TG and TC accumulation in plasma and liver. MEA or RA intake dose-dependently decreased TG and TC levels in plasma (*p <* 0.05). At equal dose, MEA and RA exhibited similar lipid-lowering effects in plasma (*p* > 0.05). In liver, MEA or RA intake dose-dependently reduced TG content, and MEA was greater than RA at equal dose in decreasing TG deposit (*p <* 0.05). MEA also dose-dependently lowered hepatic TC level (*p <* 0.05). RA at both doses, without dose-dependent manner, reduced hepatic TC level (*p <* 0.05). Histological data agreed that diabetes caused fat depositions in liver, determined by ORO stain (Figure 3). MEA or RA intake markedly decreased hepatic lipid droplets. 

### 3.3. MEA Attenuated Oxidative and Inflammatory Stress in the Heart and Kidneys

MEA intake dose-dependently lowered ROS and GSSG levels, increased GSH content and maintained the activity of GR and catalase in heat and kidney of diabetic mice (Table 4, *p <* 0.05). MEA only at high dose restored GPX activity in both organs (*p <* 0.05). RA intake only at high dose decreased ROS level in both organs (*p <* 0.05). As shown in Table 5, MEA intake dose-dependently lowered IL-1β, IL-6, TNF-α, and MCP-1 levels in the heart and kidneys of diabetic mice when compared with diabetic control groups (*p <* 0.05). RA at 0.1% also decreased cardiac and renal levels of these inflammatory factors (*p* < 0.05). RA at 0.1% exhibited similar effects as MEA at 0.5% (*p* > 0.05).

**Table 4:** Levels of ROS (RFU/mg protein), GSSG (nmol/mg protein), GSH (nmol/mg protein) and activity (U/mg protein) of GPX, GR and catalase in heart and kidney from normal (non-diabetic), diabetic mice consumed normal diet (DM), or 0.05%, 0.1% MEA or RA for six weeks. Data are mean ± SD, *n* = 10.

	Normal	DM	DM + MEA, 0.05	DM + MEA, 0.1	DM + RA, 0.05	DM + RA, 0.1
Heart						
ROS	0.19 ± 0.06 ^a^	1.45 ± 0.12 ^d^	1.14 ±0.07 ^c^	0.73 ± 0.1 ^b^	1.37 ± 0.05 ^d^	1.07 ± 0.08 ^c^
GSSG	0.21 ± 0.05 ^a^	1.09 ± 0.13 ^d^	0.85 ± 0.09 ^c^	0.49±0.11 ^b^	1.02 ± 0.07 ^d^	0.98 ± 0.06 ^d^
GSH	19.7 ± 1.3 ^d^	11.6 ± 0.8 ^a^	13.7 ± 0.5 ^b^	17.0 ± 0.4 ^c^	12.0 ± 0.9 ^a^	12.3 ± 0.8 ^a^
GPX	33.8 ± 1.0 ^c^	18.4 ± 1.5 ^a^	19.0 ± 1.2 ^a^	24.8 ± 1.1 ^b^	18.1 ± 1.5 ^a^	19.2 ± 1.2 ^a^
GR	31.4 ± 0.9 ^d^	20.8 ± 1.4 ^a^	24.2 ± 0.8 ^b^	27.8 ± 1.1 ^c^	21.0 ± 1.3 ^a^	21.4 ± 0.9 ^a^
Catalase	29.1 ± 1.4 ^d^	14.8 ± 1.1 ^a^	18.7 ± 0.6 ^b^	23.0 ± 1.3 ^c^	14.5 ± 0.9 ^a^	15.1 ± 1.0 ^a^
Kidney						
ROS	0.22 ± 0.08 ^a^	1.28 ± 0.15 ^d^	0.93 ±0.08 ^c^	0.64 ± 0.06 ^b^	1.23 ± 0.13 ^d^	0.95 ± 0.1 ^c^
GSSG	0.18 ± 0.05 ^a^	1.17 ± 0.11 ^d^	0.82 ±0.1 ^c^	0.58 ± 0.05 ^b^	1.15 ± 0.07 ^d^	1.04 ± 0.07 ^d^
GSH	14.2 ± 1.1 ^d^	5.5 ± 0.6 ^a^	7.6 ± 0.4 ^b^	10.7 ± 0.8 ^c^	6.0 ± 0.7 ^a^	6.3 ± 0.5 ^a^
GPX	21.8 ± 1.7 ^c^	14.0 ± 0.9 ^a^	14.8 ± 1.3 ^a^	18.0 ± 1.2 ^b^	14.4 ± 1.1 ^a^	14.7 ± 0.9 ^a^
GR	22.4 ± 1.0 ^d^	11.4 ± 1.1 ^a^	13.7 ± 0.9 ^b^	18.9 ± 1.4 ^c^	12.0 ± 0.6 ^a^	11.8 ± 1.3 ^a^
Catalase	20.4 ± 1.2 ^d^	12.8 ± 0.7 ^a^	14.8 ± 1.1 ^b^	17.8 ± 1.1 ^c^	13.1 ± 1.0 ^a^	15.2 ± 0.8 ^b^

^a–d^ Means in a row without a common letter differ, *p* < 0.05.

**Table 5:** Levels (pg/mg protein) of IL-1β, IL-6, TNF-α and MCP-1 in heart and kidney from normal (non-diabetic), diabetic mice consumed normal diet (DM), or 0.05%, 0.1% MEA or RA for six weeks. Data are mean ± SD, *n* = 10.

	Normal	DM	DM + MEA, 0.05	DM + MEA, 0.1	DM + RA, 0.05	DM + RA, 0.1
Heart						
IL-1β	18 ± 4 ^a^	221 ± 24 ^d^	178 ± 16 ^c^	120 ± 14 ^b^	215 ± 10 ^d^	174 ± 9 ^c^
IL-6	12 ± 2 ^a^	275 ± 28 ^d^	200 ± 20 ^c^	119 ± 14 ^b^	266 ± 22 ^d^	205 ± 18 ^c^
TNF-α	20 ± 3 ^a^	301 ± 16 ^d^	231 ± 19 ^c^	156 ± 12 ^b^	296 ± 13 ^d^	223 ± 10 ^c^
MCP-1	13 ± 4 ^a^	173 ± 12 ^d^	130 ± 8 ^c^	87 ± 11 ^b^	170 ± 14 ^d^	141 ± 7 ^c^
Kidney						
IL-1β	15 ± 3 ^a^	243 ± 19 ^d^	198 ± 10 ^c^	120 ± 12 ^b^	238 ± 19 ^d^	202 ± 15 ^c^
IL-6	18 ± 4 ^a^	238 ± 22 ^d^	162 ± 14 ^c^	95 ± 7 ^b^	229 ± 13 ^d^	175 ± 8 ^c^
TNF-α	17 ± 2 ^a^	280 ± 25 ^d^	209 ± 13 ^c^	129 ± 9 ^b^	271 ± 17 ^d^	213 ± 11 ^c^
MCP-1	10 ± 3 ^a^	217 ± 18 ^d^	160 ± 12 ^c^	103 ± 6 ^b^	213 ± 10 ^d^	167 ± 7 ^c^

^a–d^ Means in a row without a common letter differ, *p* < 0.05.

## 4. Discussion

It is reported that total triterpenic fraction prepared from *C. asiatica* alleviated diabetic microangiopathy by improving microcirculation and decreasing capillary permeability in diabetic patients [21]. Our present study further indicated that the intake of MEA, a triterpenic component of *C. asiatica,* markedly improved glycemic control, attenuated hemostatic disorder, lowered lipid accumulation, decreased oxidative and inflammatory stress in the heart and kidneys of diabetic mice. These findings support that this compound is an anti-diabetic agent. On the other hand, RA is a triterpenic acid with a similar structure to MEA. Our data revealed that RA exhibited substantial lipid-lowering effects and mild anti-inflammatory activity in diabetic mice. However, RA intake at test doses failed to affect glycemic control, hemostatic factors, and did not alter oxidative stress in the heart and kidneys of diabetic mice. Obviously, these results could not support that RA is an active compound against diabetes. We believe that this present *in vivo* study is the first report regarding the anti-diabetic effects of MEA and RA. 

MEA intake ameliorated hyperglycemia in diabetic mice via lowering glucose level and increasing insulin level. Since glycemic control has been improved, the less water and feed intake and greater body weight in MEA-treated mice could be explained. In addition, it is highly possible that the better glycemic control from MEA treatments contributed to a decrease lipid accumulation in circulation and the liver, and declined oxidative and inflammatory stress in the heart and kidneys. Diabetes is a thrombosis-prone condition because hyperglycemia-induced ROS causes platelet dysfunction [22]. Fibrinogen is a precursor for fibrin formation and a cofactor responsible for platelet aggregation, PAI-1 is the primary physiologic inhibitor for fibrinolysis [23]. Thus, the elevated plasma fibrinogen level and PAI-1 activity favored the progression of thrombosis. On the other hand, both AT-III and protein C are important anti-coagulation factors because AT-III limits the activity of several proteases in the coagulation cascade, and protein C restrains coagulation factors like factors Va and VIIIa [24]. Our data indicated that MEA intake decreased PAI-1 activity and fibrinogen level, as well as restored AT-III and protein C activities in the circulation of diabetic mice. Consequently, MEA could promote fibrinolytic reactions and alleviate aggregation, which in turn reduced the risk of diabetes associated atherogenesis and thrombosis. These novel findings indicated that MEA, not RA, could improve glycemic control and hemostatic imbalance in diabetic subjects.

Abnormal lipid accumulation in circulation and/or organs is a typical diabetic characteristic. The excessive TG and/or TC deposit facilitates the development of complications including atherosclerosis, cardiovascular pathology, and metabolic disorders [25,26]. We found the intake of either MEA or RA effectively decreased TG and TC content in blood and liver of diabetic mice. Our histological data also demonstrated that these two pentacyclic triterpenic acids markedly lowered hepatic lipid droplets. These findings suggest that these two compounds were able to improve lipid metabolism and delay the development of dyslipidemia associated diseases. The TG- and TC-lowering effects from MEA could be partially ascribed to this compound already improving glycemic control. However, RA did not significantly elevate insulin levels. Obviously, the impressed anti-lipid effects of RA were insulin-independent. Further study is necessary to examine the mechanism of RA upon lipid synthesis and/or metabolism under diabetic condition. In addition, it seems more appropriate to develop RA as an anti-lipid or anti-obesity agent. 

Oxidative stress is another crucial contributor for the progression of diabetic cardiomyopathy and nephropathy [27,28]. Our present study found that MEA supplement effectively attenuated cardiac and renal oxidative stress via decreasing the production of ROS and GSSG, and enhanced anti-oxidative defense via increasing GSH content and restoring the activity of GPX, GR, and catalase, three antioxidant enzymes in these organs. These results indicated that MEA could provide anti-oxidative protection for these organs, which in turn declined local and/or systemic oxidative stress. GR is responsible for the conversion of GSSG to GSH. Since MEA was able to maintain the activity of GR in two organs, it was reasonable to observe the greater GSH content and less GSSG level in both organs of MEA treated mice. These findings suggest that MEA improved cardiac and renal glutathione redox cycles and enhanced anti-oxidative defense in these organs. Besides lowering ROS level, RA did not affect other anti-oxidative factors in our present study. Thus, it is hard to conclude that RA is an effective anti-oxidative agent. The increased inflammatory cytokines, IL-1β, IL-6, and TNF-α, under diabetic conditions promote the progression of endothelial dysfunctions, neutrophil accumulation, and coagulation [29,30]. MCP-1, a chemotactic factor, activates monocytes and macrophages, and recruits these innate immune cells to the sites of injury [31]. Thus, the greater cytokines and chemokine generation due to diabetes disturbed immune balance and favored the occurrence of macro- or micro-vascular diseases. We found that MEA intake decreased the release of IL-1β, IL-6, TNF-α, and MCP-1 in the heart and kidneys. These results indicated that MEA could offer anti-inflammatory protection and alleviate inflammatory stress for these organs under diabetic conditions. 

MEA is a pentacyclic triterpenic acid naturally occurring in some plants foods [6]. Based on the natural property and our experimental findings, this compound could be considered as a nutraceutical agent with multiple benefits for diabetic prevention and/or attenuation. However, further study is necessary to examine the molecular mechanisms of this pentacyclic triterpenic acid. RA was a weaker anti-inflammatory compound when compared with MEA, but, it had substantial lipid-lowering effects like MEA. Thus, further research regarding the improvement of RA in lipid disorders and/or inflammatory injury is still worthy.

In summary, madecassic acid intake improved glycemic control and hemostatic imbalance in diabetic mice. Furthermore, this compound provided lipid-lowering effects in circulation and liver, and exhibited anti-oxidative and anti-inflammatory activities in hearts and kidneys of diabetic mice. Therefore, the supplement of this agent or foods rich in this compound might be helpful for the prevention or alleviation of diabetic complications.
